# Ultra-endurance race performance and muscle mass as predictors of cognitive capacity

**DOI:** 10.3389/fcogn.2025.1649669

**Published:** 2025-08-22

**Authors:** Deina Vilite, Diana Lismane, Jelena Raudeniece, Linda Laizane, Gita Gersone, Ilze Barone, Ilze Justamente, Kristiana Kovtuna, Bart Roelands, Maksims Zolovs, Dace Reihmane

**Affiliations:** ^1^Laboratory of Sports and Nutrition Research, Riga Stradinš University, Riga, Latvia; ^2^Department of Human Physiology and Biochemistry, Riga Stradinš University, Riga, Latvia; ^3^Human Physiology and Sports Physiotherapy Research Group, Faculty of Physical Education and Physiotherapy, Vrije Universiteit Brussel, Brussels, Belgium; ^4^BruBotics, Vrije Universiteit Brussel, Brussels, Belgium; ^5^Institute of Life Sciences and Technology, Daugavpils University, Daugavpils, Latvia; ^6^Statistics Unit, Riga Stradinš University, Riga, Latvia

**Keywords:** endurance exercise, cognitive functions, muscle mass, executive functions, reaction time, Stroop Test, race pacing

## Abstract

**Introduction:**

This study investigated which objective measures best predict cognitive performance in ultra-endurance athletes.

**Methods:**

Forty-three recreational cyclists completed a race of ?341 km over 18 h, with cognitive function assessed using a three-stage Stroop Test before, immediately after, and 24 h post-race. For each stage, results from the three time points were combined and analyzed using regression analyses to examine their relationship with various anthropometric, metabolic, and performance-related parameters.

**Results:**

Higher average race speed was associated with improved simple reaction time, while longer race duration predicted better performance in more cognitively demanding tasks, such as inhibitory control. Muscle mass was the only anthropometric variable significantly linked to enhanced cognition. Other factors-including age, percentage of heart rate reserve, training volume, fat mass, lean mass, bone mass, and metabolic markers-showed no associations.

**Conclusion:**

These findings suggest that race pacing and muscle mass play key roles in supporting cognitive resilience during prolonged physical exertion.

## 1 Introduction

Ultra-endurance exercise presents a unique physical challenge that tests the limits of both physiological capacity and mental resilience. While its physical demands are well-documented, the cognitive implications of such extreme exertion remain less understood. Some studies have reported impairments in complex cognitive functions post-race, while performance on simpler tasks remained stable (De Pauw et al., 2024). Others have found no change in executive functions (Krokosz et al., 2020). However, only a few studies have explored which individual characteristics may influence these cognitive outcomes.

A self-planned ultra-endurance event offers a valuable framework to explore the bidirectional brain-body relationship. Cognitive capacity can affect pacing (Sakalidis et al., 2021), perception of physical effort, and subsequent performance when exercise-induced mental fatigue sets in (Van Cutsem et al., 2017). At the same time, physical exertion can affect cognitive performance through mechanisms such as altered cerebral blood flow or heightened attention (Mulser and Moreau, 2023; Dunsky et al., 2017). Investigating these physical and mental trade-offs in a high-functioning, non-clinical population could be of particular interest, as it may facilitate performance optimization and training strategies while allowing early detection of overtraining. Additionally, studying overall cognitive function—instead of focusing specifically on before-versus-after race changes—allows for an estimation of the long-term impact of endurance exercise, muscle mass, and metabolic health.

Beyond extreme endurance events, there is robust evidence that regular physical activity benefits cognitive functioning across all age groups, with even more advantages seen in older adults (Zhang et al., 2023a). Improvements in executive function, attention, and memory are consistently observed regardless of exercise type, while global cognitive functions appear to benefit most from regular endurance training (Zhang et al., 2023a). Higher levels of cardiovascular and respiratory fitness have been linked to better cognitive outcomes both at rest and following physical exertion (Loprinzi et al., 2023). Conversely, insufficient recovery or overtraining may impair performance, including slower reaction times and reduced capacity for complex cognitive tasks (Symons et al., 2023). Furthermore, both duration and intensity of a single bout of exercise impact cognitive functions through their influence on mental fatigue. These factors increase cognitive load, resulting in higher perceived exertion and diminished cognitive performance (Van Cutsem et al., 2017).

Current research, however, highlights that cognitive performance can vary pre- and post-ultra-endurance event. Some studies report a significant post-race decline in both simple and choice reaction times, possibly due to transient alterations in cardiovascular function following prolonged exertion in healthy adults (Perrotta et al., 2022). In contrast, other studies have observed improved post-race performance in the first stage of the ST, with no significant changes in the subsequent stages compared to pre-race values (Krokosz et al., 2020). Due to these inconsistencies in the literature, the present study investigated how race performance metrics relate to cognitive performance assessed at multiple time points around an ultra-endurance event and then analyzed as a combined outcome.

Unsurprisingly, cognitive performance is linked to several factors reflecting participants' health and lifestyle prior to an ultra-endurance event. In this study, we focus on glycemic control, muscle mass, and age, as these variables have shown profound associations with cognitive functions in previous research (Casagrande et al., 2021; Wang et al., 2024; Hartshorne and Germine, 2015). Moreover, they can vary greatly among recreationally trained individuals, thus better representing the diversity seen among general population.

To highlight their relevance, poor glycemic control—indicated by elevated glycated hemoglobin (HbA1c) levels—has been consistently associated with reduced executive functions and processing speed in both healthy and clinical populations (Casagrande et al., 2021). Skeletal muscle, as a key site for insulin-mediated glucose uptake, is critical for glycemic control, hence, higher relative muscle mass is essential for maintaining healthy glucose homeostasis. Whether through impaired glucose regulation or other mechanisms, lower muscle mass has been associated with reduced cognitive performance in older adults (Wang et al., 2024).

In addition to the age-related decline in muscle mass and the corresponding rise in metabolic diseases, there is a well-documented decline in fluid intelligence beginning in the third decade of life (Hartshorne and Germine, 2015). Regular physical activity remains one of the most effective strategies for preserving muscle and preventing metabolic disease (Belzunce et al., 2023), thereby offering a means to maintain cognitive capacity throughout the lifespan.

The primary aim of this study is to examine how race performance metrics, prior training volume, age, body composition, and HbA1c levels are associated with cognitive function in the context of ultra-endurance exercise. We hypothesize that elevated HbA1c, older age, lower muscle mass, reduced resting metabolic rate (RMR), and lower training volume—either independently or in combination—will be associated with more pronounced cognitive impairment following ultra-endurance exercise.

## 2 Materials and methods

### 2.1 Study design and participants

The study was conducted during the ultra-distance cycling race, Veloreality. Held annually, the event took place from July 21–23, 2023, drawing recreational cyclists of all ages and fitness levels. Participants were required to reach all designated checkpoints by following a self-planned route, aiming to finish in the shortest time possible but no later than 42 h after the official start at 10 p.m. Consequently, the total distance covered varied, with an average of (341 ± 8 km). Forty-three of the recruited participants−39 men (91%) and 4 women (9%)–met the eligibility criteria for this study by completing the race before the 24 h mark. Participant characteristics are presented in Table 1.

**Table 1:** Descriptive statistics of the anthropo-physiological and race performance parameters.

**Parameter**	**Mean ±SD**
Age, years	39 ± 8
Finish time, min	1,098 ± 169
Average race speed, km/h	21 ± 3
Percentage of HRR, %	56 ± 7
Training volume, km	3,278 ± 1884
HbA1c, %	5 ± 0
Weight, kg	84 ± 12
Fat mass, kg	13 ± 6
Muscle mass, kg	68 ± 8
Lean mass, kg	71 ± 9
Bone mass, kg	4 ± 0
RMR, kcal	2,066 ± 263

SD, standard deviation; HRR, heart rate reserve; HbA1c, glycated haemoglobin; RMR, resting metabolic rate.

Race regulations included the prohibition of crossing private property and cycling on highways outside populated areas, unless the highway had to be crossed. Participants were allowed to rest, refuel, and sleep during the race, if they completed the race within the designated time frame. Both the starting and finishing points were in Sigulda, Latvia, a city known for its varied terrain and elevation changes.

This study was conducted in accordance with the Declaration of Helsinki and was approved by the Riga Stradinš University Research Ethics Committee (reference number: 2-PEK-4/332/2023). All participants provided written informed consent after receiving information about the study's objectives and potential risks.

### 2.2 Measures and procedures

#### 2.2.1 Anthropometric measurements

Anthropometric data—weight, fat mass, muscle mass, lean mass, and bone mass—were collected 1 to 2 days prior to the race. Body composition and RMR were assessed using a Tanita Multi-Frequency Body Composition Analyzer (MC-180MA) based on bioelectrical impedance analysis. Participants were instructed to follow the manufacturer's recommendations for pre-assessment preparation to ensure accurate and reliable measurements: (1) proper hydration should be maintained and alcohol avoided for 24 h prior to the measurement; (2) caffeine should be avoided for 12 h; (3) food intake should be refrained from for 3 to 4 h; (4) exercise should be avoided for 6–12 h; (5) showers and saunas should be avoided for 24 h; and (6) the application of creams or lotions to the palms and soles should be avoided on the day of the measurement. All participants self-reported adherence to these guidelines.

#### 2.2.2 Race performance and training history

Individual finish time was obtained from the organizing team of Veloreality. Race performance metrics—including average race speed and average heart rate during the race—were collected from participants' personal wearable devices.

Additional data extracted from these devices included resting heart rate, maximum heart rate, and total cycling distance covered in the 6 months before the race (training volume), which served as an indicator of each participant's physical conditioning.

The average percent of heart rate reserve (%HRR) was calculated using the formula:


$$\%HRR=(HR_{avg}-HR_{rest})÷(HR_{\max}-HR_{rest})\times 100\%$$

*HR*_*avg*_–*average heart rate during the race (beats per minute)*.

*HR*_*rest*_–*resting heart rate (beats per minute)*.

*HR*_*max*_–*maximum heart rate (beats per minute)*.

#### 2.2.3 Glycemic biomarker

To evaluate participants' long-term blood glucose metabolism, a blood sample was collected before the race. The samples were sent to E. Gulbis Laboratory (Riga, Latvia) for analysis, where HbA1c levels were measured.

#### 2.2.4 Cognitive function assessment

To assess participants' cognitive functions, the Stroop Test (ST) was performed at three different time points during the study: 1–2 days before the race (pre-race measurement), within 5–10 min after crossing the finish line (post-race measurement), and ~24 h after each participant's finish time (recovery measurement).

Each assessment was conducted under standardized conditions: participants were tested in groups of 4–6 people, seated in a quiet room, each at an individual computer provided by the research team. Researchers instructed participants on the procedure and provided one practice trial to familiarize them with the test format. The Stroop Test (ST) was administered using the SIA Exploro platform (exploro.lv).

The ST was used to evaluate participants' executive functions, such as inhibitory control and cognitive flexibility, as well as basic processes like attention and processing speed. The ST consisted of three stages: stage one—Stroop word-naming, stage two—Stroop color-naming, and stage three—Stroop color-word (Periáñez et al., 2021). In each stage, the primary measurement was the average time for correct responses.

Stage I assessed the speed of visual search by testing how quickly participants can respond to a simple stimulus. In this stage, participants were instructed to press the spacebar as soon as any word appeared on the screen.

Stage II introduced additional cognitive challenges, including selective attention and working memory (Periáñez et al., 2021). In this stage, participants were required to press the spacebar only when the word's meaning matched the color of the ink in which it was written (e.g., the word “red” written in red ink).

Stage III focused on assessing inhibitory control (Periáñez et al., 2021). In this stage, participants were instructed to press the spacebar only when the word's meaning did not match the color of the ink (e.g., the word “red” written in blue ink).

### 2.3 Statistical analysis

To account for random variability introduced by repeated measurements of the ST across three time points, Linear Mixed Models were used to examine the relationship between performance on each stage of the ST (dependent variables) and a range of predictors: age, finish time, average race speed, %HRR, training volume, fat mass, muscle mass, HbA1c, weight, lean mass, bone mass, and RMR.

To develop the regression models, both forward and backward stepwise selection methods were applied, evaluating all possible combinations and interactions. Model selection was guided by the Akaike Information Criterion. A Spearman's correlation matrix of all dependent and independent variables was also constructed to examine potential associations and inform the regression model-building process. To assess model fit, key assumptions—linearity, homoscedasticity, absence of multicollinearity, and normality of residuals—were tested. When the assumption of the residual normality was violated, robust p-values and confidence intervals were computed using a bootstrapping procedure with 1000 replications. All statistical analyses were conducted in Jamovi (version 2.5), with statistical significance set at *P* < 0.05.

## 3 Results

### 3.1 Descriptive measures

Table 1 presents the anthropometric, metabolic, race performance, and physical conditioning characteristics of the participants.

### 3.2 Cognitive functions

Table 2 presents the significant predictors for each stage of the ST, based on cognitive performance combined across the three time points. A negative estimate indicates that as the predictor increases, the average time for correct reactions in the ST decreases, suggesting faster correct response and, therefore, better cognitive performance.

**Table 2:** Results of the final regression model illustrating the relationship between ST performance and anthropo-physiological and race performance parameters.

**ST stage**	**Outcome variables**	**Predictor**	**Estimate**	**95% CI**	***P***
Stage I (Stroop word-naming)	Average time for correct reactions (ms)	Intercept	294.6	281.0 to 308.2	<0.001
		Average race speed	−2.5	−4.5 to −0.4	0.021
Stage II (Stroop color-naming)	Average time for correct reactions (ms)	Intercept	658.4	623.4 – 693.4	<0.001
		Muscle mass	−3.0	−1.0 to −5.0	0.003
Stage III (Stroop color-word)	Average time for correct reactions (ms)	Intercept	757.9	714.7 to 801.1	<0.001
		Finish time	−0.1	−0.3 to −0.01	0.038

CI, confidence interval.

In Stage I, each 1 km/h increase in average race speed was associated with a 2.5 ms decrease in correct reaction time. This finding suggests that higher cycling speed is positively associated with cognitive functioning on Stage I of the ST. Given the participants' average race speed of (20.7 ± 3.1 km/h), this translates to an approximate 16-ms difference in reaction time across one standard deviation.

In Stage II, each additional kilogram of muscle mass predicted a 3.0 ms reduction in correct reaction time. This suggests that individuals with greater muscle mass performed better on Stage II of the ST. With an average muscle mass of (67.6 ± 8.3 kg), this corresponds to an estimated 50 ms difference in reaction time across one standard deviation, which represents a substantial effect.

In Stage III, each additional minute required to complete the race was associated with a 0.1 ms decrease in correct reaction time. Although the effect size per minute is small, it becomes functionally relevant when considering the natural variability among participants. For instance, a difference of ~169 min (the standard deviation of finish time) could correspond to a nearly 17 ms improvement in reaction time.

Other parameters examined in this study—including age, %HRR, training volume, HbA1c, weight, fat mass, lean mass, bone mass, and RMR—did not show statistically significant associations with endurance exercise mediated cognitive performance.

## 4 Discussion

This study aimed to examine relationships between anthropometric, metabolic, and performance parameters and cognitive functions, as measured by the ST before, immediately after, and during recovery from an ultra-endurance cycling event. The key findings reveal that: (1) increased average race speed was associated with shorter reaction time in the Stroop word-naming task, reflecting improved simple reaction time; (2) greater muscle mass was associated with shorter correct reaction time in the Stroop color-naming task, which engages both working memory and simple reaction processes; (3) longer race completion time was unexpectedly positively associated with better cognitive performance in the Stroop color-word task, which involves conflict monitoring and executive function (Periáñez et al., 2021).

### 4.1 Physical performance metrics and cognitive functions

Our findings suggest that cognitive functions following an ultra-endurance event are more closely linked to actual race-day performance metrics—such as average race speed and finish time—than to relative physical load during the race (%HRR) or prior conditioning (training volume). Average speed and finish time reflect the athlete's real-time exertion and pacing strategy, while measures like %HRR and previous training volume are relative to the individual. %HRR, a widely used measure of cardiovascular exertion, can also be influenced by various factors—such as dehydration, ambient temperature, and body weight—making it a less precise indicator of the internal conditions affecting cognitive performance (Adams et al., 2014). Likewise, cycling distance over the past 6 months represents habitual activity but may not capture acute responses to the race-day stressors.

#### 4.1.1 Average race speed and Stroop word-naming

The direct influence of average race speed on simple reaction time may in fact be negative, as faster cycling presumably results in greater physical fatigue, which can slow reactions and reduce their consistency (Pavelka et al., 2020). Therefore, when evaluating overall cognitive function outcomes, as we do in this study, it is more appropriate to focus on stable baseline physical performance characteristics. For instance, greater aerobic capacity, commonly assessed via VO_2_max, likely enhances the ability to sustain higher speeds over extended periods. Research has shown that increased aerobic capacity is associated with shorter reaction times in 16–25-year-old national-level runners (Shivalingaiah et al., 2017). However, findings in females are more mixed; in healthy older women, no significant differences in simple or choice reaction times were observed despite their higher VO_2_max compared to controls (Whitehurst, 1991). It is worth noting though that reaction time tends to differ significantly by sex, generally favoring men (Jain et al., 2015).

Another key factor influencing race speed is participants' pacing strategy, which is at least partially determined by cognitive skill level. Research in intellectually impaired individuals showed that optimal pacing relies on a complex interplay between executive functions and adaptive behaviours (Sakalidis et al., 2021). While pacing strategies may vary, they are similarly governed by the brain's ability to process information in coordination with peripheral physiological systems (Gibson et al., 2006). Given that simple reaction time is a fundamental measure of processing speed, it can be argued that participants with more efficient pacing strategies are likely to exhibit shorter reaction times.

Lastly, average race speed serves as an indicator of participants' competitiveness. Indeed, performance on agility tasks improves when assessed under simulated competition conditions (Zemková et al., 2013). Because agility is tightly linked to simple reaction time, more competitive individuals may therefore excel both in the race and on cognitive tests.

#### 4.1.2 Finish time and Stroop color-word

Previous research has predominantly found a positive association between inhibitory control and athletic performance. For example, studies using the Stop-Signal Task have shown that superior inhibitory control correlates with better sporting outcomes and tends to improve with increasing levels of athletic expertise (Hagyard et al., 2021). Although the Stop-Signal Task and ST assess distinct components of response inhibition, findings using the ST similarly suggest that elite athletes, such as professional cyclists, exhibit better performance on the modified incongruent color-word Stroop task compared to recreational athletes and show greater resistance to mental fatigue (Martin et al., 2016).

In contrast, our findings reveal a different pattern. Comparing the cognitive demands of Stage I and Stage III of the ST suggests that they may tap into opposing abilities. Stage I measures simple processing speed, whereas Stage III evaluates the capacity to inhibit automatic responses. This divergence may imply that individuals with faster simple reaction times might struggle more with inhibitory control, and vice versa. In the context of race performance, this could mean that participants who cycled faster—potentially favoring a more intuitive, automatic style of decision-making—excelled in tasks requiring quick reactions but not necessarily in those demanding cognitive restraint. Conversely, participants who adopted a more deliberate and cautious pacing strategy may have demonstrated enhanced inhibitory control.

To our knowledge, no studies have directly compared simple reaction time and inhibitory control in an ultra-endurance setting. It is also important to note that inhibitory control encompasses more than just response suppression; it involves self-regulation and discipline, focused attention (e.g., ignoring interference), and cognitive inhibition (e.g., filtering intrusive thoughts) (Diamond, 2013). Given these complexities, the interplay between cognitive performance and endurance strategies warrants further investigation.

### 4.2 Muscle mass and cognitive performance

Recent studies suggest that muscle quality—not merely quantity—plays a key role in cognitive performance. By analyzing event-related potentials—small voltage fluctuations in response to specific stimuli recorded via electroencephalography—researchers have been able to more precisely and objectively assess neural activity underlying attention, cognitive inhibition, working memory, and related processes (Sur and Sinha, 2009). Notably, muscle mass and muscle strength are both associated with executive functions, but they appear to influence different cognitive domains. Muscle mass has been more closely linked to inhibitory control and conflict detection, as reflected in the N2 component of event-related potentials (Sur and Sinha, 2009; Baumgartner and Kao, 2024). In contrast, muscle strength is associated with selective attention, memory processing, and decision-making, as indexed by the P3 component (Sur and Sinha, 2009; Baumgartner and Kao, 2024). These associations have been observed independently of aerobic fitness (Baumgartner and Kao, 2024). While our study did not assess muscle strength, making direct comparison difficult, it is worth noting that muscle mass and strength are generally correlated—though this relationship weakens with age and is not strictly linear (Riviati and Indra, 2023).

Given that muscle mass has been connected to interference suppression, (Baumgartner and Kao, 2024). it is unclear why the current study did not find a significant association in Stage III of the ST, which primarily evaluates this function (Periáñez et al., 2021). Conversely, muscle mass did correlate with Stage II, which is still thought to reflect inhibitory control—albeit to a much lesser extent (Periáñez et al., 2021). This suggests that muscle mass may have a more pronounced effect on attentional processing, rather than on highly demanding conflict-monitoring tasks per se. To our knowledge, no studies have directly compared the complex interaction between muscle mass and cognitive performance across these specific cognitive domains, possibly due to their inherent overlap. Further research is warranted to elucidate the underlying mechanisms of this phenomenon.

An alternative perspective involves the endocrine role of skeletal muscle. During contraction, muscle tissue releases several myokines, including brain-derived neurotrophic factor (BDNF) and insulin-like growth factor 1 (IGF-1). BDNF has been strongly implicated in exercise-induced improvements in memory and learning (Vaynman et al., 2004). Regular endurance training elevates resting BDNF levels, which has been shown to increase hippocampal volume in both healthy adults and individuals with schizophrenia after three months of aerobic training (Pajonk et al., 2010). Given that the hippocampus is central to episodic and semantic memory, (Eichenbaum, 2004). enhanced hippocampal plasticity may partly explain exercise-related cognitive benefits, although its role in executive function is likely indirect.

A potentially more direct mechanism involves IGF-1. Exercise enhances IGF-1 expression, which supports neurogenesis and neuroplasticity while also exerting anti-inflammatory effects—mechanisms particularly relevant for protecting against cognitive dysfunction, especially in individuals with obesity (He et al., 2023).

As it relates to the current setting, the interplay between ultra-endurance cycling, muscle mass, and cognitive function has not yet been studied, though research on associations between any two of these factors does exist. For example, compared to the sedentary population, ultra-endurance athletes demonstrate increased total volumes of grey and white matter, while also showing reduced regional volumes in several brain areas involved in executive control, among other functions (Paruk et al., 2020). More research is needed to determine whether these are positive structural adaptations—such as increased neural efficiency from prolonged repetitive training—or negative consequences of chronic stress or overtraining. At the same time, while ultra-endurance exercise may alter brain volume, prolonged cycling in particular does not appear to decrease skeletal muscle mass (Knechtle et al., 2009). This is especially relevant given that sarcopenia—defined as the loss of muscle mass, strength, and function (Santilli et al., 2014)—is correlated with cognitive impairments (Hu et al., 2022).

### 4.3 Other parameters and cognitive functions

Among the additional variables considered in this study, HbA1c levels were also hypothesized to be associated with cognitive performance. Since impaired glucose metabolism also affects the brain, it can lead to reduced synthesis of neurotransmitters, impaired synaptic plasticity, and eventual neuronal damage, all of which contribute to poor cognitive performance (Zhang et al., 2023b). Chronic hyperglycemia has been shown to impair executive functions across various age groups and ethnicities, with both the prevalence and severity of cognitive deficits increasing as the disease progresses (Zhao et al., 2020).

As exercise is a vital tool in glycemic control, the majority of our study participants were normoglycemic and maintained well-regulated glucose levels. This likely eliminates the risk of cognitive decline due to glucose dysregulation, providing a plausible explanation for why HbA1c did not emerge as a significant variable in the analysis.

Age was another variable explored for its relationship to cognitive function, as it is a well-established predictor of cognitive performance at rest (Harada et al., 2013). Considering that the ST primarily assesses fluid intelligence—such as processing speed and executive function—but also involves elements of crystallized intelligence, like vocabulary recognition, due to its word-based format, these factors may shift peak performance to a later age, potentially aligning with the average age of our participant group (Harada et al., 2013).

Given that age-related cognitive decline is typically gradual in early adulthood and accelerates later in life, the subtle changes expected in our relatively young and middle-aged sample may have been too small to detect. It can also be hypothesized that age has a more pronounced effect on chronic cognitive changes, whereas acute impairments—as measured in this study—may be less sensitive to age-related effects (Harada et al., 2013). Furthermore, participants were predominantly well-trained and active, likely benefiting from neuroprotective effects. This may have delayed age-related cognitive declines, reducing the observable impact of age on performance in our study (Bliss et al., 2021).

### 4.4 Practical applications

Coaches and athletes can leverage insights into pacing strategies to tailor training and race-day approaches according to the cognitive demands of their sport, whether emphasizing reaction time or inhibitory control. Since muscle mass emerged as a key anthropometric predictor of cognitive performance, incorporating resistance training may help support cognitive resilience during endurance events. Given the limited influence of physiological measures like %HRR and HbA1c, future research should explore the relationships between cognitive capacity and these markers in diverse athletic populations and conditions.

### 4.5 Limitations

Limitations of this study include a relatively small sample size, which may have reduced the ability to detect subtle cognitive changes and limited the strength of observed associations. The use of dual-energy X-ray absorptiometry (DXA) could have improved the precision of anthropometric measurements.

Additionally, variability in HbA1c appeared to be very limited (SD not exceeding 0.5%), with mean value falling within the healthy range—likely due to the inclusion criterion requiring participants to complete the grueling race within 24 h, which is improbable for individuals in poor metabolic health.

### 4.6 Conclusions

The current study revealed that a higher average race speed during an ultra-endurance cycling event was associated with shorter simple reaction time, whereas a lower race speed was linked to better inhibitory control. Among the anthropometric measures, only muscle mass demonstrated a significant association with cognitive performance: participants with greater muscle mass exhibited superior control of working memory and selective attention.

Other variables—including age, exercise intensity (measured by %HRR), previous training volume, HbA1c, weight, fat mass, lean mass, bone mass, and RMR—did not show statistically significant associations with any of the assessed cognitive domains, as measured by the ST.

## Data Availability

The raw data supporting the conclusions of this article will be made available by the authors, without undue reservation.

## References

[B1] Adams W. M. Ferraro E. M. Huggins R. A. Casa D. J. (2014). Influence of body mass loss on changes in heart rate during exercise in the heat: a systematic review. J. Strength Cond. Res. 28, 2380–2389. 10.1519/JSC.0000000000000501 24736771

[B2] Baumgartner N. W. Kao S. C. (2024). Size or strength? How components of muscle relate to behavioral and neuroelectric measures of executive function independent of aerobic fitness. Brain Cogn. 175:106139. 10.1016/j.bandc.2024.106139 38364518

[B3] Belzunce M. A. Henckel J. Laura A. D. Horga L. M. Hart A. J. (2023). Mid-life cyclists preserve muscle mass and composition: a 3D MRI study. BMC Musculoskelet. Disord. 24:209. 10.1186/s12891-023-06283-3 36941610 PMC10026522

[B4] Bliss E. S. Wong R. H. X. Howe P. R. C. Mills D. E. (2021). Benefits of exercise training on cerebrovascular and cognitive function in ageing. J. Cereb. Blood Flow. Metab. 41, 447–470. 10.1177/0271678X20957807 32954902 PMC7907999

[B5] Casagrande S. S. Lee C. Stoeckel L. E. Menke A. Cowie C. C. (2021). Cognitive function among older adults with diabetes and prediabetes, NHANES 2011–2014. Diabetes Res. Clin. Pract. 178:108939. 10.1016/j.diabres.2021.108939 34229005 PMC8429258

[B6] De Pauw K. Ampe T. Arauz Y. L. A. Goekint M. Van Cutsem J. Roelands B.. (2024). Backyard running: pushing the boundaries of human performance. Eur. J. Sport Sci. 24, 1432–1441. 10.1002/ejsc.12190 39276329 PMC11451558

[B7] Diamond A. (2013). Executive functions. Annu. Rev. Psychol. 64, 135–168. 10.1146/annurev-psych-113011-143750 23020641 PMC4084861

[B8] Dunsky A. Abu-Rukun M. Tsuk S. Dwolatzky T. Carasso R. Netz Y.. (2017). The effects of a resistance vs. an aerobic single session on attention and executive functioning in adults. PLoS ONE. 12:e0176092. 10.1371/journal.pone.0176092 28441442 PMC5404838

[B9] Eichenbaum H. (2004). Hippocampus: Cognitive processes and neural representations that underlie declarative memory. Neuron. 44, 109–120. 10.1016/j.neuron.2004.08.028 15450164

[B10] Gibson A. C. Lambert E. V. Rauch L. H. G. Tucker R. Baden D. A. Foster C.. (2006). The role of information processing between the brain and peripheral physiological systems in pacing and perception of effort. Sports Med. 36, 705–722. 10.2165/00007256-200636080-00006 16869711

[B11] Hagyard J. Brimmell J. Edwards E. J. Vaughan R. S. (2021). Inhibitory control across athletic expertise and its relationship with sport performance. J. Sport Exerc. Psychol. 43, 14–27. 10.1123/jsep.2020-0043 33383568

[B12] Harada C. N. Natelson Love M. C. Triebel K. L. (2013). Normal cognitive aging. Clin. Geriatr. Med. 29, 737–752. 10.1016/j.cger.2013.07.002 24094294 PMC4015335

[B13] Hartshorne J. K. Germine L. T. (2015). When does cognitive functioning peak? The asynchronous rise and fall of different cognitive abilities across the life span. Psychol. Sci. 26, 433–443. 10.1177/0956797614567339 25770099 PMC4441622

[B14] He Y. Wang Q. Wu H. Ma Y. Li Z. Sun Q.. (2023). The role of IGF-1 in exercise to improve obesity-related cognitive dysfunction. Front. Neurosci. 17:1229165. 10.3389/fnins.2023.1229165 37638322 PMC10447980

[B15] Hu Y. Peng W. Ren R. Wang Y. Wang G. (2022). Sarcopenia and mild cognitive impairment among elderly adults: The first longitudinal evidence from CHARLS. J. Cachexia Sarcopenia Muscle. 13, 2636–2645. 10.1002/jcsm.13081 36058563 PMC9745544

[B16] Jain A. Bansal R. Kumar A. Singh K. D. A. (2015). comparative study of visual and auditory reaction times on the basis of gender and physical activity levels of medical first year students. Int. J. Appl. Basic Med. Res. 5, 124–127. 10.4103/2229-516X.157168 26097821 PMC4456887

[B17] Knechtle B. Wirth A. Knechtle P. Rosemann T. (2009). An ultra-cycling race leads to no decrease in skeletal muscle mass. Int. J. Sports Med. 30, 163–167. 10.1055/s-0028-1104585 19199212

[B18] Krokosz D. Bidzan-Bluma I. Ratkowski W. Li K. Lipowski M. (2020). Changes of mood and cognitive performance before and after a 100 km nighttime ultramarathon run. Int. J. Environ. Res. Public Health. 17:8400. 10.3390/ijerph17228400 33202782 PMC7697638

[B19] Loprinzi P. D. Roig M. Tomporowski P. D. Javadi A. H. Kelemen W. L. (2023). Effects of acute exercise on memory: considerations of exercise intensity, post-exercise recovery period and aerobic endurance. Mem. Cognit. 51, 1011–1026. 10.3758/s13421-022-01373-4 36401115 PMC9676734

[B20] Martin K. Staiano W. Menaspà P. Hennessey T. Marcora S. Keegan R.. (2016). Superior inhibitory control and resistance to mental fatigue in professional road cyclists. PLoS ONE. 11:e0159907. 10.1371/journal.pone.0159907 27441380 PMC4956323

[B21] Mulser L. Moreau D. (2023). Effect of acute cardiovascular exercise on cerebral blood flow: a systematic review. Brain Res. 1809:148355. 10.1016/j.brainres.2023.148355 37003561

[B22] Pajonk F. Wobrock T. Gruber O. Scherk H. Berner D. Kaizl I.. (2010). Hippocampal plasticity in response to exercise in schizophrenia. Arch. Gen. Psychiatry. 67, 133–143. 10.1001/archgenpsychiatry.2009.193 20124113

[B23] Paruk T. Rauch L. Jankiewicz M. Van Breda K. Stein D. J. King M.. (2020). Structural brain differences between ultra-endurance athletes and sedentary persons. Sports Med. Health Sci. 2, 89–94. 10.1016/j.smhs.2020.05.004 35784180 PMC9219350

[B24] Pavelka R. Trebický V. Trebická Fialová J. Landová E. Havlíček J. (2020). Acute fatigue affects reaction times and reaction consistency in mixed martial arts fighters. PLoS ONE. 15:e0227675. 10.1371/journal.pone.0227675 32004350 PMC6994193

[B25] Periáñez J. A. Lubrini G. García-Gutiérrez A. Ríos-Lago M. (2021). Construct validity of the Stroop color-word test: influence of speed of visual search, verbal fluency, working memory, cognitive flexibility, and conflict monitoring. Arch. Clin. Neuropsychol. 36, 99–111. 10.1093/arclin/acaa034 32514527

[B26] Perrotta A. S. Jeklin A. T. Bredin S. S. D. Warburton D. E. R. (2022). Effect of an ultra-endurance event on cardiovascular function and cognitive performance in marathon runners. Front. Physiol. 13:838704. 10.3389/fphys.2022.838704 35514331 PMC9065716

[B27] Riviati N. Indra B. (2023). Relationship between muscle mass and muscle strength with physical performance in older adults: a systematic review. SAGE Open Med. 11:20503121231214650. 10.1177/20503121231214650 38033420 PMC10683395

[B28] Sakalidis K. E. Burns J. Van Biesen D. Goosey-Tolfrey V. L. Gabbett T. J. Polman R.. (2021). The impact of cognitive functions and intellectual impairment on pacing and performance in sports. Psychol. Sport Exerc. 52:101840. 10.1016/j.psychsport.2020.101840

[B29] Santilli V. Bernetti A. Mangone M. Paoloni M. (2014). Clinical definition of sarcopenia. Clin. Cases Miner. Bone Metab. 11, 177–180. 10.11138/ccmbm/2014.11.3.177 25568649 PMC4269139

[B30] Shivalingaiah J. Vernekar S. S. Naik A. S. Shetty S. B. Shubha R. (2017). Aerobic capacity in runners and controls, its correlation with audio visual reaction time. Int. J. Basic Appl. Physiol. 6:87. 10.5455/njppp.2016.6.0410604062016

[B31] Sur S. Sinha V. K. (2009). Event-related potential: an overview. Indian Psychiatry J. 18, 70–73. 10.4103/0972-6748.57865 21234168 PMC3016705

[B32] Symons I. K. Bruce L. Main L. C. (2023). Impact of overtraining on cognitive function in endurance athletes: a systematic review. Sports Med. Open. 9:69. 10.1186/s40798-023-00614-3 37552398 PMC10409951

[B33] Van Cutsem J. Marcora S. De Pauw K. Bailey S. Meeusen R. Roelands B.. (2017). The effects of mental fatigue on physical performance: a systematic review. Sports Med. 47, 1569–1588. 10.1007/s40279-016-0672-0 28044281

[B34] Vaynman S. Ying Z. Gomez-Pinilla F. (2004). Hippocampal BDNF mediates the efficacy of exercise on synaptic plasticity and cognition. Eur.J. Neurosci. 20, 2580–2590. 10.1111/j.1460-9568.2004.03720.x 15548201

[B35] Wang Y. Mu D. Wang Y. (2024). Association of low muscle mass with cognitive function and mortality in USA seniors: Results from NHANES 1999–2002. BMC Geriatr. 24:420. 10.1186/s12877-024-05035-9 38734596 PMC11088051

[B36] Whitehurst M. (1991). Reaction time unchanged in older women following aerobic training. Percept.Mot.Skills. 72, 251–256. 10.2466/pms.1991.72.1.251 2038520

[B37] Zemková E. Vilman T. Kováčiková Z. Hamar D. (2013). Reaction time in the agility test under simulated competitive and noncompetitive conditions. J. Strength Cond. Res. 27, 3445–3449. 10.1519/JSC.0b013e3182903222 23524364

[B38] Zhang M. Jia J. Yang Y. Zhang L. Wang X. (2023a). Effects of exercise interventions on cognitive functions in healthy populations: a systematic review and meta-analysis. Ageing Res. Rev. 92:102116. 10.1016/j.arr.2023.102116 37924980

[B39] Zhang S. Zhang Y. Wen Z. Li C. Liu Y. Liu Y.. (2023b). Cognitive dysfunction in diabetes: abnormal glucose metabolic regulation in the brain. Front. Endocrinol. 14:1192602. 10.3389/fendo.2023.1192602 37396164 PMC10312370

[B40] Zhao Q. Zhang Y. Liao X. Wang W. (2020). Executive function and diabetes: a clinical neuropsychology perspective. Front. Psychol. 11:2112. 10.3389/fpsyg.2020.02112 32973635 PMC7468478

